# Identification of Stripe Rust Resistance Genes in Common Wheat Cultivars and Breeding Lines from Kazakhstan

**DOI:** 10.3390/plants10112303

**Published:** 2021-10-26

**Authors:** Alma Kokhmetova, Aralbek Rsaliyev, Angelina Malysheva, Makpal Atishova, Madina Kumarbayeva, Zhenis Keishilov

**Affiliations:** 1 Institute of Plant Biology and Biotechnology, Almaty 050040, Kazakhstan; malysheva_angelina@list.ru (A.M.); maki_87@mail.ru (M.A.); madina_kumar90@mail.ru (M.K.); jeka-sayko@mail.ru (Z.K.); 2 Research Institute of Biological Safety Problems, Gvardeiskiy 080409, Kazakhstan; aralbek@mail.ru

**Keywords:** wheat, *Triticum aestivum* L., stripe rust, *Puccinia striiformis*, resistance genes, molecular markers

## Abstract

Stripe (yellow) rust, caused by *Puccinia striiformis* f.sp. *tritici* (*Pst*), is a fungal disease that presents one of the most serious threats to the wheat crops, causing severe yield losses worldwide, including Kazakhstan. The objectives of this study were to: (1) evaluate a winter wheat collection for stripe rust resistance during an adult plant growth stage, (2) identify the presence of selected *Yr* genes using linked molecular markers in wheat germplasm, (3) identify potentially useful resistant wheat genotypes among leading cultivars and advanced breeding lines. This study evaluated 70 winter wheat genotypes for stripe rust resistance. According to the field reactions, 42 entries (60%) had R or MR reactions including 27 breeding lines (38.6%) and 15 (21.4%) cultivars. Twenty-eight breeding lines/cultivars (40.0%) were susceptible in both years. According to the average coefficient of infection value (ACI) six genotypes were regarded as possessing high level of adult plant resistance. Cultivars/lines carrying *Yr10* alone or in combination with other *Yr* resistance genes provided resistance to stripe rust. Eleven breeding lines showed <5% disease severity in both years. Linked marker analysis revealed the presence of several gene and gene complexes (*Yr5*, *Yr10*, *Yr15*, *Yr17/Lr37/Sr38* and *Yr18/Lr34*). Among a collection of 70 winter wheat breeding lines and cultivars produced in Kazakhstan three stripe rust resistance genes (*Yr10*, *Yr5* and *Yr15*) demonstrated high frequency occurrence (31.4%, 14.0% and 7.0%, respectively). The most abundant was gene *Yr10* identified in 22 genotypes. It was followed by the *Yr5* gene, which conferred resistance in 14 lines (20%) and *Yr18* gene-11 lines (15.7%). *Yr15* was identified in 7 genotypes. *Yr17/Lr37/Sr38* gene complex was found in 2 entries. Among 70 evaluated germplasm sources, 42 disease resistant entries are potentially useful resistant wheat genotypes. These carriers of different *Yr* genes can be used directly in breeding programs to improve stripe rust resistance of winter wheat. Marker-assisted selection can be efficiently applied to develop wheat cultivars with effective gene combinations that would directly assist in developing durable resistance in Kazakhstan.

## 1. Introduction

World grain production has been increasing in recent years, but the global loss of wheat from diseases is 10% of the potential harvest (FAOSTAT data. http://www.fao.org/faostat/en/#data/QC, accessed on 23 November 2016). Wheat, *Triticum aestivum L.*, is the most cultivated cereal crop, and is directly linked to food security. One of the most serious threats to the wheat crops is fungal diseases comprising of rusts causing severe yield losses worldwide [1]. Wheat production in Kazakhstan is seriously constrained due to rust diseases, including stem rust caused by *Puccinia graminis* f. sp. *tritici* Eriks., stripe rust caused by *Puccinia striiformis* Westend. f. sp. tritici and leaf rust caused by *Puccinia triticina* Erikss [2,3,4,5,6,7,8], as well as leaf spot diseases (tan spot and Septoria) [9,10,11,12,13,14].

Stripe, or yellow rust, *Puccinia striiformis*, is one of the most widespread and damaging diseases of wheat in Kazakhstan [3,15]. Stripe rust infection can occur anytime from one-leaf stage to plant maturity, provided that plants are still green [16]. This disease reduces the photosynthetic capacity, increases transpiration, and reduces the accumulation of organic matter, resulting in shriveled grain with low quality. Stripe rust of wheat has been reported in more than 60 countries and in all continents except Antarctica [16]. In recent years, major wheat producing countries have faced severe stripe rust epidemics leading to significant yield losses [1,17]. The capability of the pathogen for mutation and rapid generation turnover accelerates the development of races. The airborne spread of inoculum can reach a distance of hundreds of kilometers. Yahyaoui (2003) suggests that the main mechanisms of pathogen evolution in Central Asia are represented by a sequence of mutations and genetic recombination [18].

There have been frequent occurrences of stripe rust epidemics in many parts of the region of Central Asia and Kazakhstan [6,19,20]. Epiphytotic development of stripe rust caused by an abnormal amount of precipitation leads to severe crop reduction. In Kazakhstan, stripe rust attacked many commercial wheat cultivars causing severe infection in Kazakhstan. In 2002, it caused 30–40% loss of yield in the southeast region [2]. The stripe rust severities on winter wheat have substantially increased between 2001 and 2010 in Central and West Asia [21]. Continuous outbreaks of stripe rust in recent years are also reflected through the occurrence of four epidemics in different parts of Central Asia in 2009–2014 [20,22,23].

Disease caused serious damage to both yield and quality. Recent studies have shown that 20 to 40% of wheat grain yield losses could occur due to stripe rust on the susceptible varieties [24]. The breakdown of resistance in commercial cultivars suggests that the conventional selection improves resistance slowly. One reason for slow progress is the limited effectiveness of the selection technique to identify the presence of multiple resistance genes in breeding lines before releasing for commercial cultivation [6]. Additionally, a set of pathogen isolates that could help identify multiple resistance genes is not established for stripe rust in Central Asia. Hence, application of known molecular markers linked to stripe rust resistance genes could accelerate efforts to improve resistance.

To date, according to the Catalogue of Gene Symbols, more than 80 genes with official or temporary symbols for resistance to stripe rust have been identified [25]. However, the resistance effectiveness against stripe rust can be overcome by emergence of new pathogen races. Most of these genes are dominant, race-specific and therefore, do not provide durable resistance independently. Therefore, identification of novel sources of resistance in a cultivar is of foremost importance for effective disease control. 

Only a few *Yr* genes such as *Yr5* and *Yr15* are effectively resistant to all known *Pst* races in China and around the world [26]. Diversity in *Yr* genes in commercial cultivars could play an important role in managing frequent stripe rust epidemics in the region. Kokhmetova et al. (2010) reported that the most effective resistant genes against stripe rust in Kazakhstan are *Yr2*+, *Yr4*+, *Yr5*, *Yr10* and *Yr15* [15]. Additionally, there are a few *Yr* genes that confer non-race-specific resistance, acting at the adult plant stage such as *Yr18*, which is a multi-pathogen resistance gene and confers part field resistance against stripe rust, leaf rust, stem rust, and powdery mildew have been used in breeding programs for a century and so far, no pathogen adaptability has been found [27]. 

The stripe rust resistance gene *Yr5* was originally identified in hexaploid *Triticum aestivum* ssp. spelta var *album* (TSA). Macer (1966) located it on chromosome 2B and Law (1976) further localized it to the long arm of that chromosome, 21 cM from the centromere, is a race-specific R-gene effective at both seedling and all plant growth stages and located on the chromosome 2BL [28,29]. *Yr5* confers resistance to almost all isolates of *P. striiformis tritici* in the world, except for Australia [30] and India [31]. *Yr5* confers resistance to almost all isolates of *P. striiformis tritici* in Kazakhstan [6]. 

The dominant stripe rust resistance gene *Yr10* was originally found in wheat line PI 178383 and cultivar Moro [32] and located on chromosome 1BS, 2 cM apart from Rg1 locus that confers brown glume colour [33] and 5 cM from locus Gli-1B [34]. This gene has been mapped in different loci on 1 and 6 groups of wheat chromosomes. Bariana et al., 2002 verified close association between Yr10 and Gli-B1 by genetic analysis of the cultivar Moro [35]. The Yr10 resistance gene continues to that provide effective resistance to stripe rust in many parts of the world. This gene has been reported effective against all races in China [32], India [36], Pakistan [37], Iran [38], USA [39] and in Kazakhstan [15]. 

The dominant stripe rust resistance gene *Yr15* was identified in *Triticum dicoccoides* accession G-25 [40] and mapped on chromosome 1BS [41]. Currently, the main all-stage resistance genes which are used in breeding programs and are effective against all currently identified races in the U.S. are *Yr5*, *Yr15* and *Yr45* [42]. *Yr15* confers broad-spectrum resistance against a worldwide collection of more than 3000 genetically diverse *Pst* isolates, including modern races, such as ‘Warrior’ (race DK09/11), which is currently threatening wheat production [43]. Our previous studies have shown that *Yr15* is still effective against stripe rust isolates Kazakhstan [3].

The *Yr17*, *Lr37* and *Sr38* rust resistance genes, which confer resistance in wheat against stripe, leaf and stem rust, have been used by breeders in different parts of the world [44,45]. These linked resistance genes are located in a 2NS/2AS translocation [46]. Rust races with virulence to *Yr17* and *Lr37* have been identified in different countries but this gene cluster still provides resistance to a wide range of races and is useful in combination with other rust resistance genes [47].

The locus *Lr34/Yr18/Pm38* confers partial and durable resistance against the devasting fungal pathogens leaf rust, stripe rust, and powdery mildew. *Yr18/Lr34* genes have been used in breeding programs for a century and so far, no pathogen adaptability has been found [27]. The gene complex *Yr18/Lr34* are known as “slow rusting gene” which provides durable and non-specific APR and located on the short arm of chromosome 7D. The high durability of *Yr18/Lr34* explains by the race unspecific adenosine triphosphate-binding cassette-transporter [48]. *Yr18/Lr34* is expressed in adult plants during the critical grain-filling stage and is most effective in the flag leaf. Wheat cultivars containing these genes occupy more than 26 million ha in various developing countries alone and contribute substantially to yield savings in epidemic years [49].

Phytopathological methods based on symptomology are not always effective for the identification of resistance genes. Field evaluation is expensive, time-consuming and highly affected by environmental conditions. A diversified and effective resistant gene resource must be the basis of breeding wheat cultivars with rust resistance. Incorporation of the resistance genes is an eco-friendly system that does not place any cost burden on the growers. Nowadays, various molecular markers have been widely used in plant genetic mapping and marker-assisted selection (MAS). Molecular markers associated with disease resistance will be a more effective way to identify disease resistance factors. The advent of relatively inexpensive, high throughput molecular marker platforms results in marker-assisted selection (MAS) becoming a viable approach to tracking resistance genes [26].

The objectives of this study were to: (1) evaluate a winter wheat collection for stripe rust resistance during the adult plant growth stage, (2) identify the presence of selected *Yr* genes using linked molecular markers in wheat germplasm, (3) identify potentially useful resistant wheat genotypes among leading cultivars and advanced breeding lines.

## 2. Results

### 2.1. Field Evaluation of Adult Plant Resistance

Uniform and consistent stripe rust development was observed for adult plant resistance in the field evaluation. ANOVA showed significant differences among genotypes (*p* < 0.001) to the stripe rust severity in both growing season (Table S1). Stripe rust severities in both years (2019 and 2020) are shown in Table 1. Stripe rust development varied greatly among the wheat entries. According to the field reactions, 42 entries (60.0%) had R and MR reactions including 29 breeding lines (41.4%) and 13 (18.6%) cultivars. Among 70 genotypes 22 breeding lines (31.4%) and 6 cultivars (8.6%) were susceptible in in both years. Eleven breeding lines (1777Darya/Tungysh-2, 12-12/1613MP-2011/1027/AVS/Ulugbek/Egemen, 1017/103F3/N91/5353/ Egemen, Rils Almaly/Anza, 5-ICARDA-IPBB-2013, 5221/Almaly, Naz/GF55, #23/Kupava-7, #23/Kupava-12, #23/Kupava-24) showed <5% disease severity in both years.

### 2.2. Identification of Yr Genes with Molecular Markers and Stripe Rust Resistance in the Sources of Resistance

Linked marker analysis revealed the presence of several genes and gene complexes (Table 1). Detection of five *Yr* genes in wheat genotypes was carried out using 9 *Yr* gene linked markers. 

Three sequence tagged site (STS) markers *S19M93* [50], *S23M41* [50], and *STS-9/10* [51]. Linked with yellow rust resistance gene *Yr5* were used to confirm these markers in wheat genotypes. *S19M93-140* located at 0.54 cM from *Yr5* amplified one 100 bp allele, and *S23M41* amplified one 275 bp allele. Both *S19M93* and *S23M41* are closely linked to *Yr5* gene and these markers have been reported as co-segregating with *Yr5* gene [50]. Another STS marker, *STS-9/10*, developed by Chen et al. (2003), co-segregate with the *Yr5* locus, located at 0.7 cM from *Yr5* and amplified fragments of 439 or 433 bp for the resistant or susceptible plants, respectively [51]. These molecular markers were chosen to screen 70 cultivars/lines for the presence of *Yr5* sources. Screening with *S19M93* marker produced the expected 100 bp band associated with *Yr5* gene in 18 genotypes (25.7%), as well as in the control line Avocet S*6/Yr5 (Cat# 4, 9, 13, 14, 20, 33, 34, 44, 46, 47, 50, 51, 58, 63, 66, 67, 69) (Figure S1). The other 52 wheat genotypes (74.3%) failed to amplify the gene. As an example, the PCR results using *S19M93* marker are shown in the Figure S1. STS marker *S23M41* amplified product size 275 bp linked to Yr5 gene was observed in 14 entries (27.1%) (Cat# 4, 9, 13, 14, 20, 33, 34, 44, 46, 58, 63, 66, 67, 69) (Figure S2). STS marker, STS-9/10 was chosen to screen wheat entries for the presence of *Yr5* sources. The plant materials amplified fragments of 439 or 433 bp for the resistant or susceptible plants, respectively. Polymorphism was better revealed after *Dpn*II digestion of the PCR products. The *Yr5* sources had bands with sizes 289 bp, while non-carriers of *Yr5* amplified 182 bp PCR products (Figure S3). Fifteen genotypes (Cat# 3, 4, 9, 13, 14, 20, 33, 34, 44, 46, 47, 58, 63, 66, 67, 69) amplified the same band as isogenic line Avocet S*6/Yr5 (Table 1). As a result of confirming the presence of *Yr5* with all three *Yr5* linked markers, 14 genotypes (20%) were identified as carriers of *Yr5* (Cat# 4, 9, 13, 14, 20, 33, 34, 44, 46, 58, 63, 66, 67, 69).

The gene *Yr10* identified in 22 genotypes. Marker *Xpsp3000* located on the end of chromosome 1BS linked with the stripe rust resistant gene, *Yr10*, with a distance 1.2 cM [32]. The fragment 260 bp was a specific band closely linked to the stripe rust resistance gene *Yr10*. The microsatellite marker *Xpsp3000* is inherited in a co-dominant manner, and can be used to identify genotypes of individuals at any growth stage [52] for marker assisted selection of *Yr10* [32,35]. Considering the above, the *Xpsp3000* marker is suitable for the identification of resistant genotypes at different stages of plant development. Using this marker, the fragment 260 bp was amplified in tested entries. The molecular marker linked to *Yr10*, *Xpsp3000* amplified fragment size 260 bp in 22 genotypes (Cat# 5, 7, 13, 23, 28, 30, 32, 33, 34, 36, 37, 39, 42, 45, 46, 52, 54, 58, 60, 61, 65, 68), while the remaining 48 genotypes were lacking in *Yr10* (Figure S4, Table 1). In addition to the SSR *Xpsp3000* marker, the SCAR marker linked to *Yr10* with a genetic distance of 0.5 cM [53] was chosen to screen the 70 entries in our study. The fragment 200 bp was a specific band closely linked to the stripe rust resistance gene *Yr10*. The fragment 180 bp was a non-specific band and was amplified in most of the tested wheat materials. The positive control, Avocet S*6/Yr10 showed 200bp band, and susceptible check Avocet S a 180 bp band (Table 1, Figure S5). Twenty-three genotypes (Cat# 5, 7 13, 23, 28, 30, 32, 33, 34, 36, 37, 38, 39, 42, 45, 46, 52, 54, 58, 60, 61, 65, 68), amplified the same band as *Yr10* carrier isogenic line. In addition to 23 homozygous entries, four segregating for *Yr10* locus (Cat# 47, 48, 49, 51) have been detected. Total, approximately 38%) of all 70 entries (27 genotypes) assayed with SCAR marker in this study were predicted to possess *Yr10.* (Figure S5, Table 1). Wheat genotypes that confirmed the presence of this gene as a result of PCR with both *Xpsp3000* and *Yr10SCAR* markers were assigned to carriers of the *Yr10* gene. On this basis, 22 genotypes (31,4%) (Cat# 5, 7, 13, 23, 28, 30, 32, 33, 34, 36, 37, 39, 42, 45, 46, 52, 54, 58, 60, 61, 65, 68) carried *Yr10* gene.

Molecular markers linked to the *Yr15* gene were identified by Sun et al. (1997), Peng et al. (2000) and Murphy et al. (2009) [54,55,56]. The *Yr15* gene was mapped to a 6.4 cM interval flanked by marker *Xbarc8*, located 3.9 cM to the distal side, and by *Xgwm413* located 2.5 cM to the proximal side. It was determined that *Xbarc8* and *Xgwm413* are diagnostic of the *Yr15* gene across almost all backgrounds tested [56]. In the present study these two markers were used to screen among the 70 entries for *Yr15* detection. *Xbarc8* marker amplified 2 alleles (257 and 221 bp). The expected size of the fragment amplification for locus *Xbarc8* coupled to the resistant allele of *Yr15* gene was 221 bp (Figure S6, Table 1). Out of 70 genotypes tested for *Yr15* the expected PCR product was amplified in eight genotypes (Cat# 3, 29, 30, 40, 41, 42, 50, 53), including seven homozygous entries and one genotype segregating for *Yr15* locus. The fragment 257 bp was a non-specific band and was amplified in the rest 62 wheat entries. Screening with *Xgwm413* marker produced the expected 96 bp band associated with the *Yr15* gene in seven genotypes (Figure S7, Table 1). On the basis of confirmation of *Yr15* with both *Xbarc8* and *Xgwm413* markers, seven genotypes (10%) (Cat# 3, 29, 30, 40, 41, 42, 53) carried the *Yr15* gene.

The presence of the *Yr1*7 gene in wheat genotypes was studied using the *VENTRIUP/LN2* marker is associated with *Triticum ventricosum* chromosome 2NS translocated to the short arm of bread wheat chromosome 2AS [47]. Of the 70 cultivars/lines identified to carry these resistance genes in our study, two genotypes (114Novosibirskaya-22/Omskaya37/28 and 425/Renan) amplified 262-bp fragment, indicating the presence of the *Yr17/Lr37/Sr38* resistance gene block (Figure S8, Table 1).

A specific co-dominant STS marker *csLV34*, which is a bi-allelic locus, was used to detect the presence/absence of the gene complex *Yr18/Lr34*. Genetic linkage between *csLV34* and *Yr18/Lr34* was estimated at 0.4 cM [57]. The robustness of the *csLV34* marker in postulating the likely occurrence of *Lr34/Yr18* across a wide range of germplasm was earlier confirmed [52]. The 150-bp and 229-bp bands indicated the presence and absence of the *Yr18* gene, respectively. Marker analyses indicate that amplification products correspond with the homozygous resistant allele of *Lr34/Yr18* gene were detected in 11 wheat entries (Cat# 2, 3, 9, 13, 20, 35, 54, 58, 62, 64, 70), accounting for 15.7% of studied genotypes (Figure S9, Table 1).

Wheat germplasm was classified into four groups according to ACI value. Wheat entries with ACI values of 0–10, 11–20, 21–30, 31–60 were regarded as possessing high, moderate-resistant, moderate-susceptible and low levels of adult plant resistance. The 2-year average ACI values for the entries ranged from 3.44 and 8.04 for the 1st (boot stage) and the 2nd (milk stage) observations, respectively. Details of ACI data in wheat genotypes (carriers of *Yr* genes) are presented in Table S2.

The ACI values of susceptible control Morocco and Avocet S reached 55.0 and 90.0 which shows high disease pressure. The study of resistance isogenic lines Avocet S*6/Yr5, Avocet S*6/*Yr10*, Avocet S*6/*Yr5* indicated that resistance genes *Yr5*, *Yr10* and *Yr15* showed a high level of resistance to stripe rust during two growing seasons (0–1.5 ACI values during the 2 years). Resistance of *Yr17* and *Yr18* genes was lower (ACI values 5–14 and 1.5–6) for two genes, respectively (Table S2).

Out of 42 carriers of *Yr* genes 35 wheat entries possessed ACI value of 0–20 conferred high and moderate resistant level of adult plant resistance, seven had ACI regarded as possessing either MS or S reactions.

The most resistant cultivars (0 and 10.0 average ACI values) were 30 entries. Six genotypes (#23/Kupava-7, 5-ICARDA-IPBB-2013, Naz/GF55-2, #23/Kupava-12, #23/Kupava-24, #23/Kupava-5) were highly resistant (ACI values: 0 and 1.0); four of them carrying *Yr10* alone or in combination with *Yr5* or *Yr15*. The line #23/Kupava-12 consist *Yr5* alone, wihle #23/Kupava-5 had *Yr15* gene.

Amongst the next 24 resistant genotypes (ACI value: 2–10) three entries possessing the 3 *Yr* genes were detected: 114Novosibirskaya-22/Omskaya37/28 (*Yr5*, *Yr17*, *Yr18*), while Mereke-70 and 1777Darya/1724F1-1581/807F4 /Naz/Umanka/Almaly/Zimorodok-1 both combining *Yr5*, *Yr10*, *Yr18* genes. In three entries: Taza/MK 3750-2 (*Yr*, *Yr10*), 1010/93f3/N23/Kupava/Mereke70-1 (*Yr5*, *Yr18*) and Karasay (*Yr10*, *Yr18*), two *Yr* genes in each were found. The rest of the genotypes had one *Yr* gene each. In a group of five genotypes with a moderately resistant reaction (ASI 12 for the 2nd observation), four entries had the *Yr10* gene, and the Almaly cultivar had only one gene (*Yr18*).

Cultivars/lines carrying *Yr10* alone or in combination with other *Yr* resistance genes in most cases provided high level of resistance against stripe rust (Table S2). The genotypes with three *Yr* genes (*Yr5*, *Yr17*, *Yr18* and *Yr5*, *Yr10*, *Yr15*) showed low ACI (2–7) and were regarded as possessing high level of resistance.

According to the ACI value *Yr18* gene provided high level of resistance in seven lines, but four entries (Almaly/GF70, 425/GF55-1, Kupava and Almaly/GF92), carrying this gene demonstrated MS and S reactions to stripe rust. (Table S2). Analysis of ACI data indicates that the effectiveness of *Yr17/Lr37/Sr38* depended on the genetic background and the combination of genes. In one genotype 114Novosibirskaya-22/Omskaya37/28 presence of this gene complex in the combination with *Yr5* and *Yr18/Lr34* provided a high level of resistance, but in the line 425/Renan, a *Yr17/Lr37/Sr38* susceptible reaction against stripe rust was observed.

Thus, one or more *Yr* gene and gene complex were detected in 42 genotypes, including 27 breeding lines and 15 cultivars of wheat. Several resistant genotypes did not possess any of the five *Yr* gene and gene complex. As a rule, genotypes identified as resistant under field conditions, had one or more *Yr* gene. The *Yr10* gene can be considered the most effective in the studied germplasm, which provided a highly resistant and moderately resistant level of resistance in 22 wheat genotypes (31.4%). It was followed by the *Yr5* gene, which conferred resistance in 14 lines (20%) and the *Yr18* gene-11 lines (15.7%). Among 70 evaluated germplasm sources, 42 disease resistant entries were potentially useful resistant wheat genotypes.

## 3. Discussion

Rust diseases are the perennial problem for winter wheat in Central Asia and the Caucasus [58]. Evaluation stripe rust resistance includes artificially inoculated greenhouse experiments and both artificially and naturally infected field experiments. These methods can be time consuming, are limited to the growing season, and require maintenance of the various stripe rust races to detect specific genes for resistance [51,59]. In addition, races of *P. striiformis* that differentiate *Yr5* and *Yr15* have not been identified so it is currently not possible to use greenhouse or field resistance phenotyping to identify genotypes possessing both resistance genes, unless they are identified using progeny testing and test crosses. Alternatively, as for many traits, marker-assisted selection (MAS) is an efficient tool to select for and predict phenotype [56].

The study of winter wheat germplasm allowed to evaluate the promising lines and cultivars for genetic and breeding programs aimed at improving stripe rust resistance of wheat in Kazakhstan. In this study, 51 promising wheat breeding lines, developed by the Institute of Plant Biology and Biotechnology, as well as 19 widely grown cultivars in Kazakhstan were tested with linked markers for some *Yr* genes. These 70 wheat genotypes greatly differ in stripe rust severity recorded at adult plant stage in the field. Eleven breeding lines showed <5% disease severity in both years, suggesting their potential value as sources of resistance. However, several genotypes (Naz/GF55-2, Sanzar8/BWKLDN9, 1777Darya/Tungysh-1, 1777Darya/Tungysh-3, 1017/103F3/N91/5353/Egemen-1, and 1011/94F3/N23/Knyazhna/Naz-1) showed high stripe rust severity in both years, suggesting their low potential value as sources of resistance. None of the five genes were detected in a few highly resistant genotypes (1777Darya/Tungysh-2, 12/1613MP-2011/1027/AVS/Ulugbek/Egemen, 1017/103f3/N91/5353/Egemen-1, Rils Almaly/Anza and 5221/Almaly) suggesting that additional *Yr* genes are conferring resistance in these genotypes. This demonstrates the diversity of *Yr* genes in the gene pool comprising of recently released cultivars and advanced breeding lines of winter wheat in Kazakhstan.

Molecular markers are useful for identifying lines with multiple genes and for pyramiding multiple resistance genes, which is difficult and sometimes impossible to do using only phenotypic data [60]. In different previous studies, the sources of *Yr* resistance genes (*Yr9*, *Yr5*, *Yr10*, *Yr15* and *Yr18* genes) were identified in winter wheat breeding material [37,61,62]. The result of a study by Zheng et al. (2017) showed effective resistance of *Yr15* and *Yr65* genes; significant additive effects were observed in some gene combinations, such as *Yr9*+*Yr18* and *Yr30*+*Yr46* [63].

Deployment of specific gene combinations provides durable and improved resistance versus using single genes because a single specific gene is subject to become susceptible due to genetic shifts in the pathogen [64]. In our study, nine closely linked markers specific for 5 *Yr* genes were used to detect the presence of *Yr5*, *Yr10*, *Yr15*, *Yr17* and *Yr18* genes among the wheat genotypes and evaluate their effect on the resistance to stripe rust. For better accuracy of results, the *Yr5* gene was amplified by using three linked markers, and *Yr10* and *Yr15*–by using two linked markers. The combined results of all the markers were used for confirming the presence of *Yr* genes.

In our research, molecular screening of spring wheat cultivars showed contrasting differences in the frequencies of five important *Yr* genes (*Yr5*, *Yr10*, *Yr15*, *Yr17* and *Yr18*). Three genotypes (114Novosibirskaya-22/Omskaya37/28, Mereke-70 and1777Darya/1724F1-1581/807F4 /Naz/Umanka/Almaly/ Zimorodok-1) were identified with maximum 3 *Yr* genes followed by 2 *Yr* genes in eight genotypes (#23/Kupava-7, Naz/GF55-2, #23/Kupava-24, Taza/MK 3750-2, 1010/93f3/N23/Kupava/Mereke70-1, Karasay, Naz/GF55-3 and 425/GF55). These genotypes exhibited resistance response at the field.

Among a collection of 70 winter wheat breeding lines and cultivars produced in Kazakhstan three stripe rust resistance genes (*Yr10*, *Yr5* and *Yr15*) demonstrated high frequency occurrence, 31.4%, 20.0% and 10%, respectively. These stripe rust resistant genes showed evidence of providing adequate protection in the investigated wheat entries (<20% disease severity). This supports previous reports on varietal resistance [20] and that the improved stripe rust resistant winter wheat germplasm is increasingly becoming available in Central Asia [6,23].

In terms of the resistance spectrum, *Yr5* has broad-spectrum resistance to stripe rust [30,65] and confers resistance to almost all isolates of *P. striiformis tritici* in the world, except for Australia [30] and India [31]. *Yr5* is found to be effective against all rust virulent races in North America [51,66], Iran [67], China [16], India [31,36], Turkey [68] and Kazakhstan [6]. As a result of our research 14 genotypes (20%) were identified as carriers of *Yr5*. As *Yr5* is a race-specific seedling resistance gene, it should be used in combination with other effective genes and/or with race non-specific adult-plant resistance genes. Such a combination could provide durable resistance [69]. In our studies, the *Yr5* gene provided a high level of protection against the pathogen in combination with the *Yr10* (Cat # 27,15, 31,7,16), with the *Yr18* (# 6, 9, 30, 31, 7) and in combination with APR genes *Yr17* and *Yr18* (# 6).

The *Yr10* gene is found race specific and has been reported effective against all races in China [32], Iran [67], Pakistan and USA [66]. No virulence for *Yr10* has been found also in Kazakhstan [15]. In the present study, *Yr10* was detected in 22 cultivars/lines. Previously, the *Yr10* gene was postulated in the Adir cultivar as a result of a genetic segregation analysis [15]. In this study using marker analysis the previous finding was confirmed in the cv Adir, as well as in the line with this cultivar in their pedigree (Adir/*Yr2*). The presence of *Yr10* was confirmed in the breeding lines derived from the spliting populations. For instance, from the population Naz/GF55 three lines (Naz/GF55-2 Naz/GF55-3 and Naz/GF55-4) were selected as the carriers of *Yr10;* the source of this gene was cv Naz, confirmed in a previous study [70]. The same results were obtained in the line #46 (#23/Kupava-24) with identified *Yr10*, in the pedigree of which the presence of this gene was confirmed. Earlier, in the line #23 [Brundage/Naz/Mereke70], three stripe rust resistant genes (*Yr5*, *Yr10*, *Yr18*) were identified (Annual report, project 2120-GF4, Kokhmetova et al., unpublished data). So, the *Yr10* gene was the most abundant in this research (31.4%).

Abundance of *Yr10* among the genotypes in this study is expected, considering that many advanced breeding lines have originated from IPBB germplasm, which have sources of the *Yr10* gene in their pedigree. These include cultivars Naz, Mereke70, Matay which are carriers of the *Yr10* gene [6]. Crosses aimed at the introduction of the *Yr5* gene, carried by *Triticum spelta album* from the IPBB collection, cultivar Zimorodok, as well as the isogenic line Avocet S*6/Yr5. This made it possible to develop breeding lines carrying the *Yr5* gene. Seven advanced breeding lines carrying the *Yr15* gene were developed on the basis of crossing the target wheat germplasm with the isogenic line Avocet S*6/Yr15. The breeding line 1777Darya/1724F1-1581/807F4/Naz/Umanka/Almaly/Zimorodok-1 and cultivar Mereke70 had the highest number of resistance genes (*Yr5*, *Yr10* and *Yr18*). Field evaluation of breeding material demonstrated that these three genes are effective. The resistance of five breeding lines was due to the two gene combinations: 1010/93F3/N23/Kupava/Mereke70-1 (*Yr5*, *Yr18*), 114Novosibirskaya-22/Omskaya37/28 (*Yr5*, *Yr17*), #23/Kupava-24 (*Yr5*, *Yr10*), Naz/GF55-2 (*Yr10*, *Yr15*), and #23/Kupava-7 (*Yr10*, *Yr15*). Among race specific genes *Yr5*, *Yr10* and *Yr15* are still protective against current predominant races in Kazakhstan. The result obtained indicates the efficiency of incorporating the stripe rust resistance genes in breeding material.

Currently, the main all-stage resistance genes which are used in breeding programs and are effective against all identified races in the U.S. are *Yr5*, *Yr15* and *Yr45* [42]. Our previous studies have shown that genes *Yr5* and *Yr15* are also effective against stripe rust isolates in Kazakhstan [15]. On the basis of confirmation of *Yr15* with both *Xbarc8* and *Xgwm413* marker, seven genotypes (10%) carried the *Yr15* gene.

The yellow rust resistant gene *Yr17* linked to *Lr37* is still effective against yellow rust in some regions and may explain the popularity of this gene complex. Although virulence for *Lr37* has occurred in Europe and Australia, this gene is still recommended for breeding in many countries, including Russia and Kazakhstan [71,72]. The genes *Yr17* and *Yr18* were found in the current study much less frequently than *Yr10* and *Yr5*: *Yr17* and *Yr18* were detected at a frequency of 2.8% and 11%, respectively. The results obtained by Ullah et al., 2016 indicates that most of the line applied in their study lack this alien chromatin [37]. Madenova et al. (2016) identified one genotype with *Lr34/Yr18* genes and two genotypes with complex genes *Lr37/Sr38/Yr17* [73]. Our results also showed a similar trend with these findings and *Yr18/Lr34* gene was observed in 11 genotypes and *Yr17/Lr37* in 2 genotypes.

The broad sense heritability (*h_b_*^2^) estimates for stripe rust across years were high (from 0.86 to 0.89) indicating that resistance to stripe rust can be improved by selection (Table S1). Similar heritability estimates for disease reaction have been reported by Singh et al. (2019) and Genievskaya et al. (2020) [74,75].

The molecular markers are a convenient and efficient approach to identify effective stripe rust resistance genes in cultivars and lines, and particularly so where a well-characterized pathogen collection is not available for multi-pathotype assessments. Since the evaluated entries included germplasm, coming from a breeding program directed to stripe rust resistance improvement and many crosses included CIMMYT-developed germplasm in their pedigree, they are likely to have diverse resistance gene constitutions. Among these sources, 42 disease resistant entries, which are the carriers of different *Yr* genes, can be used directly in breeding programs to improve stripe rust resistance of winter wheat. Marker-assisted selection can be efficiently applied to develop wheat cultivars with effective gene combinations that would directly assist in developing durable resistance in Kazakhstan.

## 4. Materials and Methods

### 4.1. Plant Material

This study assessed 70 winter wheat genotypes, including 19 cultivars and 51 elite breeding lines from Kazakhstan (Table 1), which were evaluated for *Puccinia striiformis* resistance in the field tests and in molecular screening for presence of *Yr* genes. This germplasm is produced or used in breeding programs of Kazakhstan. The highly susceptible control cultivar Morocco as well as the near isogenic lines (NILs) of cv. Avocet S NIL Yr5/6* Avocet S, NIL Yr10/6* Avocet S, NIL Yr15/6* Avocet S and (NILs) of cv. Thatcher: NIL Lr37 TC*6/VPM (RL 6081) and NIL Lr34 TC*6/PI58548 were also used in both tests.

### 4.2. Experimental Site

Evaluation of field resistance to stripe rust was carried out under conditions of the Kazakh Research Institute of Agriculture and Crop Production (KazNIIZiR), (Almalybak, 43°13′09″ N, 76°36′17″ E, Almaty region) in Southeast Kazakhstan, Almaty region, during 2019 and 2020 cropping seasons. Each entry was planted in 1 m^2^ plot in mid-September. Experiments were conducted with a completely randomized design with two replicates in 1 m^2^. The stripe rust susceptible cultivar Morocco was planted in every 10th row and as a spreader border around the nursery to ensure uniform infection. Fertilizer treatments, 60 and 30 kg/ha of N and P_2_O_5_, respectively, and other management practices corresponded to those normally recommended for the region [76]. Annual rainfall ranged from 332 to 644 mm during the 2 years. Experimental plants were sown in 1 m^2^ plots in mid-April every two experimental years. Weather conditions in Almaty in 2019 and in 2020 were favorable for the development of stripe rust, and the infection on susceptible checks reached 20S and 40S, respectively; however, there was a severe late development of stripe rust reaching 80% on susceptible check Morocco. So, the growing seasons were favorable for pathogen infection and disease development. Mean daily temperature and relative humidity showed similar trends in both years (Table S3). The average maximum air temperature for mid-May in 2019 and 2020 reached 31.3 and 32.5 °C, respectively. From April to June 2019, mean daily temperature was 11.4, 16.6, and 21.6 °C, respectively, and in 2020, 11.4, 16.6, and 21.8 °C. From April to June 2019, the monthly rainfalls and average relative humidity (RH) were 168, 39 and 72 mm, and 59.5%, respectively, and in 2020, 140, 74, 30 mm, and 57.3% (www.pogodaiklimat.ru/monitor.php accessed 15 June 2021)—conditions highly conducive for stripe rust infection and development.

### 4.3. Field Evaluation of Adult Plant Resistance

Infection type and severity data were recorded in late May and early June when the plots were at boot and milk stages, respectively. The time of second evaluation was also determined when rust severity on the susceptible control Morocco reached >60%. In mid-April 2019, stripe rust induced susceptible cultivars Morocco and Avocet S were inoculated with mixed races of *Pst* at seedling stage in the field in Kazakhstan to serve as spreader of stripe rust pathogen to the experimental plots. Morocco and Avocet S were planted in every 10th row and as spreader border around the nursery to ensure uniform infection. The material was screened in natural conditions and no artificial inoculation was carried out. The experiment was conducted using randomized complete block design with three replications and recommended cultural practices were used for trial management.

Scoring of stripe rust symptoms was performed according to the method developed at the CIMMYT [77]. Both infection type and severity data were recorded in late May and early June when the crops were at boot and milk stages, respectively, when severity on the susceptible check reached >60%. Five infection types described as the following: 0—immune (no uredia or other symptoms of disease infection); R—resistant (uredia minute, supported by distinct necrotic areas); MR—moderately resistant (uredia small to medium, in green islands surrounded by chlorotic tissue); MS—moderately susceptible (uredia medium in size, no necrotic but chlorotic areas may be present); and S—susceptible (uredia large, no necrosis but chlorosis may be evident). Stripe rust severities were recorded on three replications. For the replicated data means were calculated. Partial resistance in the field was evaluated at boot and milk stages accordingly, using the modified Cobb scale [78], as well as the coefficients of infection (CI) and the average coefficient of infection (ACI). CI was calculated by multiplying the severity values by the constant values for infection types, based on: R = 0.2, MR = 0.4, MS = 0.8 and S = 1.0 [79]. The genotypes showing terminal ratings ≤20% stripe rust severity was classified as resistant. ANOVA was analyzed using R statistical software (R Core Team, 2018), using replications as fixed effect and entries as random effect [80]. The least significant difference (LSD) test was used for significance of differences between the means. The broad sense heritability index, describing the proportion of phenotypic variation due to genetic factors, was calculated based on the ANOVA outcome as *h_b_^2^* = SSg/SS_t_, where SS_g_ is the sum of squares for genotype and SS_t_ is the total sum of squares (Table S1).

### 4.4. DNA Extraction and Identification of Yr Genes with Molecular Markers

A set of 70 winter wheat genotypes including released cultivars and advanced breeding lines was used in the study. The markers linked to five *Yr* genes and gene complexes (*Yr5*, *Yr10*, *Yr15*, *Lr34/Sr57/Yr18* and *Lr37/Sr38/Yr17* were used. Genomic DNA was extracted from fresh leaves of single plants at the two-leaf seedling stage for each genotype using the CTAB method [81]. The polymerase chain reaction (PCR) method was used for identification of *Yr* resistance gene carriers. Wheat genotypes in which the resistance genes had been previously identified were used as a positive control, while samples in which resistance genes had not been previously detected were used as a negative control. Specific recommended protocols were used for primers linked to different *Yr* genes. The presence of molecular markers to resistance genes *Yr5* (*S19M93*, *S23M4 Yr5STS-9/10)* [50,51], *Yr10* (Xpsp3000, *Yr10SCAR*) [32,53], *Yr15* (*Xbarc8*, *Xgwm413*) [55,82], *Lr37/Sr38/Yr17* (*Ventriup/LN2*) [57] *Lr34/Sr57/Yr18* (*csLV34*) [47] and was determined according to the procedure outlined by Smith et al. [50], Chen et al. [51], Wang et al. [32], Shao et al. [53], Peng et al. [55], Helguera et al. [47] and Lagudah et al. [57] (Table 2). The *Yr5* gene carriers were detected based on PCR using the STS-9/10 marker [51]; for this marker, 0.5 µL (5U) of restriction enzyme *Dpn*II and 1.3 µL of 10xbuffer for *Dpn*II (new England Biolabs, Beverly, MA, USA) were added to the remaining 10 µL of PCR product. Samples were incubated at 37 °C for 2 h and digestion products were separated in 2.5% (*w*/*v*) agarose gels.

Primers and annealing temperature conditions of polymerase chain reaction (PCR) were carried out as described for each *Yr* gene in the references (Table 2). PCR reactions were performed in a Bio-Rad T100TM Thermal Cycler (Bio-RAD, Hercules, California, USA). The PCR mixture (25 µL) contained 2.5 µL of genomic DNA (30 ng), 1 µL of each primer (1 pM/µL) (SigmaAldrich, St. Louis, MI, USA), 2.5 µL of dNTP mixture (2.5 mM, dCTP, dGTP, dTTP and dATP aqueous solution) (ZAO Sileks, Sayansk, Russia), 2.5 µL MgCl2 (25 mM), 0.2 µL Taq polymerase (5 units µL) (ZAO Sileks, Russia), 2.5 µL 10X PCR buffer and 12.8 µL ddH20. PCR amplification was performed with a Mastercycler (Eppendorf, Hamburg, Germany) with initial denaturation at 94 °C for 3 min, 45 cycles: 94 °C for 1 min, annealing at 60 °C for 1 min, 72 °C for 2 min and final elongation at 72 °C for 10 min. The amplification products were separated on 2% agarose gel in TBE buffer (45 mM Tris-borate, 1 mM EDTA, pH 8) [83] with the addition of ethidium bromide. To determine the length of the amplification fragment, a 100-bp DNA ladder (Fermentas, Vilnius, Lithuania) was included. Results were visualized using the Gel Documentation System (Gel Doc XR+, BIO-RAD, Hercules, CA, USA). The specific amplification procedures were in accordance with the corresponding references (Table 2). The test was repeated for each sample three times.

## Figures and Tables

**Table 1:** Disease severity to stripe rust and presence of *Yr* genes in wheat genotypes from Kazakhstan.

Cat #	Cultivar/Line Name	Origin ^a^	Yellow Rust Severity %, RT ^b^		Molecular Marker Test ^c^									
			2019	2020	*S19M93*	*S23M14*	*STS* *9/10*	*Yr10* *SCAR*	*Xpsp* *3000*	*Xbarc8*	*Xgwm413*	*csLV34*	*VENTRIUP/LN2*	*Yr* Gene Detected Based on Linked Marker
1	Naz/GF55-1	KZ:Almaty-KIZ	40S	40MS	0	0	0	0	0	0	0	0	0	*-*
2	Almaly/GF70	KZ:Almaty-KIZ	30MS	20MS	0	0	0	0	0	0	0	1	0	*Yr18*
3	425/GF55-1	KZ:Almaty-KIZ	20MS	30MS	0	0	0	0	0	1	1	1	0	*Yr15*, *Yr18*
4	Kupava/YR5/6/Avocet ‘S’	KZ:Almaty-KIZ	30MS	20MS	1	1	1	0	0	0	0	0	0	*Yr5*
5	Adir/YR2	KZ:Almaty-KIZ	10MR	10MR	0	0	0	1	1	0	0	0	0	*Yr10*
6	Sanzar8/BWKLDN9	KZ:Almaty-KIZ	50S	40S	0	0	0	0	0	0	0	0	0	–
7	Viza/Zhenis	KZ:Almaty-KIZ	10MR	10MR	0	0	0	1	1	0	0	0	0	*Yr10*
8	1777Darya/#72Tungysh	KZ:Almaty-KIZ	30MS	40MS	0	0	0	0	0	0	0	0	0	–
9	114Novosibirskaya-22/Omskaya37/28	KZ:Almaty-KIZ	10MR	0	1	1	1	0	0	0	0	1	1	*Yr5*, *Yr17*, *Yr18*
10	1777Darya/Tungysh-1	KZ:Almaty-KIZ	30MS	40MS	0	0	0	0	0	0	0	0	0	–
11	1777Darya/Tungysh-2	KZ:Almaty-KIZ	0	0	0	0	0	0	0	0	0	0	0	–
12	1777Darya/Tungysh-3	KZ:Almaty-KIZ	30S	40S	0	0	0	0	0	0	0	0	0	–
13	1777Darya/1724F1-1581/807F4/Naz/Umanka/ Almaly/ Zimorodok-1	KZ:Almaty-KIZ	15R	20MR	1	1	1	1	1	0	0	1	0	*Yr5*, *Yr10*, *Yr18*
14	1777Darya/1724F1-1581/807F4/Naz/Umanka/ Almaly/Zimorodok-2	KZ:Almaty-KIZ	10MR	0	1	1	1	0	0	0	0	0	0	*Yr5*
15	12/1613MP-2011/1027/AVS/ Ulugbek600 /Egemen	KZ:Almaty-KIZ	0	0	0	0	0	0	0	0	0	0	0	–
16	1017/103f3/N91/5353/Egemen-1	KZ:Almaty-KIZ	0	0	0	0	0	0	0	0	0	0	0	–
17	1017/103f3/N91/5353/Egemen-2	KZ:Almaty-KIZ	50S	40S	0	0	0	0	0	0	0	0	0	–
18	1011/94f3/N23/Knyazhna/Naz-1	KZ:Almaty-KIZ	30MS	40MS	0	0	0	0	0	0	0	0	0	–
19	1011/94f3/N23/Knyazhna/Naz-2	KZ:Almaty-KIZ	20MS	30MS	0	0	0	0	0	0	0	0	0	–
20	1010/93f3/N23/Kupava/Mereke70-1	KZ:Almaty-KIZ	10MR	10MR	1	1	1	0	0	0	0	1	0	*Yr5*, *Yr18*
21	1010/93f3/N23/Kupava/Mereke70-2	KZ:Almaty-KIZ	0	0	0	0	0	0	0	0	0	0	0	–
22	Rils Almaly/Anza	KZ:Almaty-KIZ	15MS	30MS	0	0	0	0	0	0	0	0	0	–
23	5-ICARDA-IPBB-2013	IWWIP-ICARDA-CIMMYT	5R	0	0	0	0	1	1	0	0	0	0	*Yr10*
24	5221/Almaly	KZ:Almaty-KIZ	0	0	0	0	0	0	0	0	0	0	0	–
25	Naz/GF66/Ulugbek600-1	KZ:Almaty-KIZ	30S	30MS	0	0	0	0	0	0	0	0	0	–
26	Naz/GF66/Ulugbek600-2	KZ:Almaty-KIZ	30S	40MS	0	0	0	0	0	0	0	0	0	–
27	Naz/Immun78/MK3750	KZ:Almaty-KIZ	10R	0	0	0	0	0	0	0	0	0	0	–
28	Almaly/YR4/Naz	KZ:Almaty-KIZ	10MR	0	0	0	0	1	1	0	0	0	0	*Yr10*
29	RILS-F9 Almaly/Avoset ‘S’	KZ:Almaty-KIZ	10MR	5MR	0	0	0	0	0	1	1	0	0	*Yr15*
30	Naz/GF55-2	KZ:Almaty-KIZ	5R	0	0	0	0	1	1	1	1	0	0	*Yr10*, *Yr15*
31	Bogarnaya56/5515/K-47100-Romania	KZ:Almaty-KIZ	70S	90S	0	0	0	0	0	0	0	0	0	–
32	Taza/MK 3750-1	KZ:Almaty-KIZ	20MR	10MR	0	0	0	1	1	0	0	0	0	*Yr10*
33	Taza/MK 3750-2	KZ:Almaty-KIZ	5MR	10MR	1	1	1	1	1	0	0	0	0	*Yr5*, *Yr10*
34	Naz/GF55-3	KZ:Almaty-KIZ	20MS	10MS	1	1	1	1	1	0	0	0	0	*Yr5*, *Yr10*
35	Almaly/GF92	KZ:Almaty-KIZ	30S	40MS	0	0	0	0	0	0	0	1	0	*Yr18*
36	428/MK-122A	KZ:Almaty-KIZ	10MR	10MS	0	0	0	1	1	0	0	0	0	*Yr10*
37	Naz/GF55-4	KZ:Almaty-KIZ	20MR	20MS	0	0	0	1	1	0	0	0	0	*Yr10*
38	425/Renan	KZ:Almaty-KIZ	30MS	50MS	0	0	0	1	0	0	0	0	1	*Yr17*
39	425/GF55-2	KZ:Almaty-KIZ	20MS	20MR	0	0	0	1	1	0	0	0	0	*Yr10*
40	Almaly/GF70/2	KZ:Almaty-KIZ	30MS	20MS	0	0	0	0	0	1	1	0	0	*Yr15*
41	#23/Kupava-5	KZ:Almaty-KIZ	10R	0	0	0	0	0	0	1	1	0	0	*Yr15*
42	#23/Kupava-7	KZ:Almaty-KIZ	0	0	0	0	0	1	1	1	1	0	0	*Yr10*, *Yr15*
43	#23/Kupava-10	KZ:Almaty-KIZ	30S	30MS	0	0	0	0	0	0	0	0	0	–
44	#23/Kupava-12	KZ:Almaty-KIZ	5R	0	1	1	1	0	0	0	0	0	0	*Yr5*
45	#23/Kupava-16	KZ:Almaty-KIZ	10MR	0	0	0	0	1	1	0	0	0	0	*Yr10*
46	#23/Kupava-24	KZ:Almaty-KIZ	5R	0	1	1	1	1	1	0	0	0	0	*Yr5*, *Yr10*
47	1010/93/#23/Kupava/ Mereke/Naz	KZ:Almaty-KIZ	0	0	1	0	1	1+0	0	0	0	0	0	*Yr10yr10*
48	807-2011/Babax1/907/Almaly 29266/Sultan2	KZ:Almaty-KIZ	30MS	10MS	0	0	0	1+0	0	0	0	0	0	*Yr10yr10*
49	Almaly/YR18	KZ:Almaty-KIZ	10R	0	0	0	0	1+0	0	0	0	0	0	*Yr10yr10*
50	1596-2#23/Kupava/ Ulugbek/YR4/Mereke/T.Spelta-YR5-1	KZ:Almaty-KIZ	5R	10MR	1	0	0	0	0	1+0	0	0	0	*Yr15yr15*
51	1596-3#23/Kupava/ Ulugbek/YR4/Mereke /T. Spelta-YR5-1	KZ:Almaty-KIZ	10MR	20MR	1	0	0	1+0	0	0	0	0	0	*Yr10yr10*
52	Adir	KG	20MR	10MR	0	0	0	1	1	0	0	0	0	*Yr10*
53	Keremet	KZ: KIZ	5R	10MR	0	0	0	0	0	1	1	0	0	*Yr15*
54	Karasay	KZ: KIZ	10R	15MR	0	0	0	1	1	0	0	1	0	*Yr10*, *Yr18*
55	Umanka	RU	40MS	30MS	0	0	0	0	0	0	0	0	0	–
56	Kyzylbiday	KZ: KIZ	40MS	30S	0	0	0	0	0	0	0	0	0	–
57	Sanzar8	UZ	70S	50S	0	0	0	0	0	0	0	0	0	–
58	Mereke70	KZ: KIZ	20MR	10MR	1	1	1	1	1	0	0	1	0	*Yr5*, *Yr10*, *Yr18*
59	Yuzhnaya12	KZ: KIZ	50S	40S	0	0	0	0	0	0	0	0	0	–
60	Matay	KZ: KIZ	20MS	20MR	0	0	0	1	1	0	0	0	0	*Yr10*
61	Naz	KZ: KIZ	10MR	20MR	0	0	0	1	1	0	0	0	0	*Yr10*
62	Nureke	KZ: KIZ	20MR	10MR	0	0	0	0	0	0	0	1	0	*Yr18*
63	Dinara	KZ:Almaty-KIZ	10MR	20MR	1	1	1	0	0	0	0	0	0	*Yr5*
64	Kupava	RU	20MS	30MS	0	0	0	0	0	0	0	1	0	*Yr18*
65	Sultan2	KZ: KIZ	10MR	5MR	0	0	0	1	1	0	0	0	0	*Yr10*
66	Tungysh	KZ: KIZ	5MR	10R	1	1	1	0	0	0	0	0	0	*Yr5*
67	Taza	KZ: KIZ	15MR	20MR	1	1	1	0	0	0	0	0	0	*Yr5*
68	Intensivnaya	KZ: KIZ	10MS	20MR	0	0	0	1	1	0	0	0	0	*Yr10*
69	Zimorodok	RU	10MR	20MS	1	1	1	0	0	0	0	0	0	*Yr5*
70	Almaly	KZ: KIZ	20MR	20MS	0	0	0	0	0	0	0	1	0	*Yr18*
71	Morocco	MAROCCO	80S	90S	0	0	0	0	0	0	0	0	0	-
71	Avocet S*6/Yr5	AUSTRALIA	5R	0	1	1	1	0	0	0	0	0	0	*Yr5*
72	Avocet S*6/Yr10	AUSTRALIA	R	10R	0	0	0	1	1	0	0	0	0	*Yr10*
73	Avocet S*6/Yr15	AUSTRALIA	5R	0	0	0	0	0	0	1	1	0	0	*Yr15*
74	YR17/LR37/NIL-LR37/TC-6/VPM-RL6081	AUSTRALIA	10MS	25MS	0	0	0	0	0	0	0	0	1	*Yr17*
75	YR18/NIL-LR34/TC-6/PI58548	AUSTRALIA	10MR	10MS	0	0	0	0	0	0	0	1	0	*Yr18*
76	Avocet S	AUSTRALIA	90S	90S	0	0	0	0	0	0	0	0	0	-

^a^—Origin includes countries and organizations: ICARDA—CAC (IWWP), KZ—Kazakhstan, KG–Kyrgyzstan, RU–Russia, KIZ–Kazakh Research Institute of Agriculture and Crop Production, Almaty–Institute of Plant Biology and Biotechnology. ^b^—Values indicate severity, RT—reaction type. ^c^—“1”, “0”, “1 + 0” and “-” indicate the presence, absence and heterozygote allele of corresponding gene, respectively.

**Table 2:** Specific primer sequences, PCR annealing temperature, expected size and references for selected *Yr-*genes.

Gene	Marker Type	Primer Name	Primer Sequence (5′-3′)	Annealing Temp. (°C)	Fragment Size (bp)	Reference
*Yr5*	STS	*S19M93*	TAATTGGGACCGAGAGACG TTCTTGCAGCTCCAAAACCT	62	100	[50]
*Yr5*	STS	*S23M41*	TCAACGGAACCTCCAATTTC AGGTAGGTGTTCCAGCTTGC	58	275	[50]
*Yr5*	STS	*Yr5STS-9/10*	AAA GAA TAC TTT AAT GAA3 CAA ACT TAT CAG GAT TAC3	60	+289 −182	[51]
*Yr10*	SSR	Xpsp3000	GCAGACCTGTGTCATTGGTC GATATAGTGGCAGCAGCAGGATAC	55	+260 −240	[32]
*Yr10*	SCAR	*Yr10SCAR*	CTG CAG AGT GAC ATC ATA CA TCG AAC TAG TAG ATG CTG GC	55	200 +180	[53]
*Yr15*	SSR	*Xbarc8*	GCG GGA ATC ATG CAT AGG AAA ACA GAA GCG GGG GCG AAA CAT ACA CAT AAA AAC A	60	+250 −280	[83]
*Yr15*	SSR	*Xgwm413*	TGCTTGTCTAGATTGCTTGGG GATCGTCTCGTCCTTGGCA	60	96	[55]
*Yr17/Lr37/Sr38*	SCAR	*VENTRIUP/LN2*	AGG GGC TAC TGA CCA AGG CT TGC AGC TAC AGC AGT ATG TAC ACA AAA	65	262	[57]
*Yr18/Lr34*	STS	*csLV34*	GTT GGT TAA GAC TGG TGA TGG TGC TTG CTA TTG CTG AAT AGT	60	+150 −229	[47]

## Data Availability

Data are contained within the article and supplementary material.

## Supplementary Materials

The following are available online at https://www.mdpi.com/article/10.3390/plants10112303/s1, Table S1: Analysis of variance (ANOVA) for stripe rust resistance and the estimated broad sense heritability, Table S2: An average coefficient of infection values (ACI) of the wheat germplasms carrying the stripe rust resistance genes (Almalybak, Almaty region, Kazakhstan, 2019 and 2020), Table S3: Mean daily temperature and relative humidity (Almalybak, Almaty region, Kazakhstan, 2019 and 2020), Figure S1: DNA amplification products of wheat entries using primers to the STS S19M93 locus linked with the *Yr5* resistance gene, Figure S2: DNA amplification products of wheat entries using primers to the STS S23M41locus linked with the *Yr5* resistance gene, Figure S3: DNA amplification products of wheat entries using primers to the STS-9/10 locus linked with the *Yr5* resistance gene, Figure S4: DNA amplification products of wheat entries using primers to the SSR Xpsp3000 locus linked with the *Yr10* resistance gene, Figure S5: DNA amplification products of wheat entries using primers to the Yr10SCAR locus linked with the *Yr10* resistance gene, Figure S6: DNA amplification products of wheat entries using primers to the SSR Xbarc8 locus linked with the *Yr15* resistance gene, Figure S7: DNA amplification products of wheat entries using primers to the SSR Xgwm413 locus linked with the *Yr15* resistance gene, Figure S8: DNA amplification products of wheat entries using primers to the Ventriup/LN2 locus linked with the *Yr17* resistance gene, Figure S9: DNA amplification products of wheat entries using primers to the STS csLV34 locus linked with the *Yr18/Lr34* resistance gene.
