# Supplementary material for: Identification of Stripe Rust Resistance Genes in Common Wheat Cultivars and Breeding Lines from Kazakhstan
Source: Plants (Basel). 2021 Oct 26;10(11):2303. doi: 10.3390/plants10112303 (PMC8619625; doi:10.3390/plants10112303)
Supplement: Supplementary file 1 [file plants-10-02303-s001.zip › plants-1402324-supplementary.pdf]

plants

MDPI

Article

Identification of Stripe Rust Resistance Genes in Common Wheat Cultivars and Breeding Lines from Kazakhstan

Alma Kokhmetova [1,*], Aralbek Rsaliyev [2], Makpal Atishova [1], Madina Kumarbayeva [1], Zhenis Keishilov [1] and Angelina Malysheva [1]

Supplementary Materials

Table S1. Analysis of variance (ANOVA) for stripe rust resistance and the estimated broad sense heritability.

	Source	df	SS	MS	F	(h_b^2)
YR 2019	Genotype	69	50015,357	724,8602	15,781**	0,89
	Replication	2	11,428	5,7142	4,124*	
	Residuals	138	6338,571	45,9317		
	Total	209	56365,357			
YR 2020	Genotype	69	61055,357	884,8602	18,27223172**	0,89
	Replication	2	517,143	258,5714	5,339*	
	Residuals	138	6682,8571	48,4265		
	Total	209	68255,3571			

* $P < 0.01$. ** $P < 0.001$. YR, yellow rust; df, degree of freedom; SS, sum of squares; MS, mean squares; h_b^2, broad-sense heritability index.

Table S2. An average coefficient of infection values (ACI) of the wheat germplasms carrying the stripe rust resistance genes (Almalybak, Almaty region, Kazakhstan, 2019 and 2020).

Cat #	Cultivar (line)	Yr gene detected based on linked marker	[a]ACI value		[b]ACI rank
			1st observation	2nd observation	
42	#23/Kupava-7	*Yr10, Yr15*	0	0	1
23	5-ICARDA-IPBB-2013	*Yr10*	0	0,5	1
30	Naz/GF55-2	*Yr10, Yr15*	0	0,5	1
44	#23/Kupava-12	*Yr5*	0	0,5	1
46	#23/Kupava-24	*Yr5, Yr10*	0	0,5	1
41	#23/Kupava-5	*Yr15*	0,5	1	1
28	Almaly/YR4/Naz	*Yr10*	0	2	1
66	Tungysh	*Yr5*	0	2	1
9	114Novosibirskaya-22/Omskaya37/28	*Yr5, Yr17, Yr18*	0,5	2	1
14	1777Darya/1724F1-1581/807 F4/Naz /Umanka/Almaly/Zimorodok-2	*Yr5*	0,5	2	1
45	#23/Kupava-16	*Yr10*	0,5	2	1
53	Keremet	*Yr15*	0,5	2,5	1
65	Sultan2	*Yr10*	0	3	1
29	RILS-F9 Almaly/Avoset 'S'	*Yr15*	0,5	3	1
33	Taza/MK 3750-2	*Yr5, Yr10*	0,5	3	1
5	Adir/YR2	*Yr10*	1	4	1
7	Viza/Zhenis	*Yr10*	1	4	1
20	1010/93f3/N23/Kupava/Mereke70-1	*Yr5, Yr18*	1	4	1

54	Karasay	*Yr10, Yr18*	2,5	4	1
58	Mereke70	*Yr5, Yr10, Yr18*	1,5	6	1
63	Dinara	*Yr5*	2	6	1
32	Taza/MK 3750-1	*Yr10*	2,5	6	1
36	428/MK-122A	*Yr10*	2,5	6	1
52	Adir	*Yr10*	2,5	6	1
61	Naz	*Yr10*	2,5	6	1
62	Nureke	*Yr18*	2,5	6	1
13	1777Darya/1724F1-1581/807F4 /Naz/Umanka/Almaly/ Zimorodok-1	*Yr5, Yr10,Yr18*	2	7	1
67	Taza	*Yr5*	2,5	7	1
68	Intensivnaya	*Yr10*	0,5	8	1
69	Zimorodok	*Yr5*	4	10	1
39	425/GF55-2	*Yr10*	2,5	12	2
34	Naz/GF55-3	*Yr5, Yr10*	4	12	2
60	Matay	*Yr10*	5	12	2
37	Naz/GF55-4	*Yr10*	6	12	2
70	Almaly	*Yr18*	10	12	2
2	Almaly/GF70	*Yr18*	4	20	2
3	425/GF55-1	*Yr15, Yr18*	10	20	2
4	Kupava/ YR5/6/Avocet'S'	*Yr5*	10	20	2
64	Kupava	*Yr18*	11	20	2
40	Almaly/GF70/2	*Yr15*	12	20	2
35	Almaly/GF92	*Yr18*	16	31	4
38	425/Renan	*Yr17*	20	32	4
		Controls			
71	Morocco	-	55	85	4
71	Avocet S*6/Yr5	*Yr5*	0	0,5	1
72	Avocet S*6/Yr10	*Yr10*	0	1,5	1
73	Avocet S*6/Yr15	*Yr15*	0	0,5	1
74	YR17/LR37/NIL-LR37/TC-6/VPM-RL6081	*Yr17*	5	14	2
75	YR18/NIL-LR34/TC-6/PI58548	*Yr18*	1,5	6	1
76	Avocet S	-	55	90	4
	Average		3,44	8,04	1,38
	LSD (p=5%)		5,91	7,96	-

[a]ACI – average coefficient of infection values for two years.
[b]ACI rank of the level of resistance based on disease score across 42 carriers of Yr genes based on 2nd observation: wheat entries having ACI values of 0-10, 11-20, 21-30, 31-60 were regarded as possessing high (R), moderate-resistant (MR), moderate-susceptible (MS)and low (S) level of adult plant resistance.

Table S3. Mean daily temperature and relative humidity s (Almalybak, Almaty region, Kazakhstan, 2019 and 2020).

Year	Month	Temperature (°C)	Monthly rainfalls (mm)	Average relative humidity (%)
	April	11,4	168	
2019	May	16,6	39	59,50
	June	21,6	72	
	April	11,4	140	
2020	May	16,6	47	57,30
	June	21,8	30	

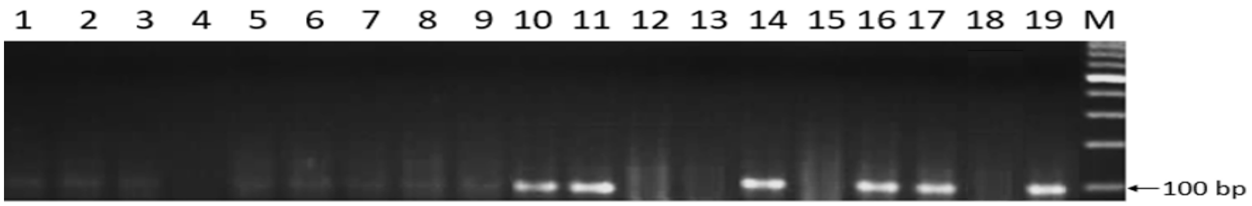

1, 5221/Almaly; 2, Naz/GF66/Ulugbek600-1; 3, Naz/GF66/Ulugbek600-2; 4, Naz/Immun78/MK3750; 5, Almaly/YR4/Naz; 6, RILS-F9 Almaly/Avoset 'S'; 7, Naz/GF55-2; 8, Bogarnaya56/5515/K-47100-Romania; 9, Taza/MK 3750-1; 10, Taza/MK 3750-2; 11, Naz/GF55-3; 12, Almaly/GF92; 13, 428/MK-122A; 14, #23/Kupava-12; 15, #23/Kupava-16; 16, #23/Kupava-24; 17, 1010/93/#23/Kupava/Mereke/Naz; 18, Avocet'S' (negative control); 19, Avocet S*6/Yr5 (positive control); M -molecular weight marker (Gene-Ruler, 100 bp DNA ladder).

Figure S1. DNA amplification products of wheat entries using primers to the STS S19M93 locus linked with the *Yr5* resistance gene. The arrows show the band size of *Yr5*-carrying germplasm (100 bp). The sizes of the bands for *Yr5* are 100 bp (lanes 10, 11, 14, 16, 17 and 19 – positive control). .

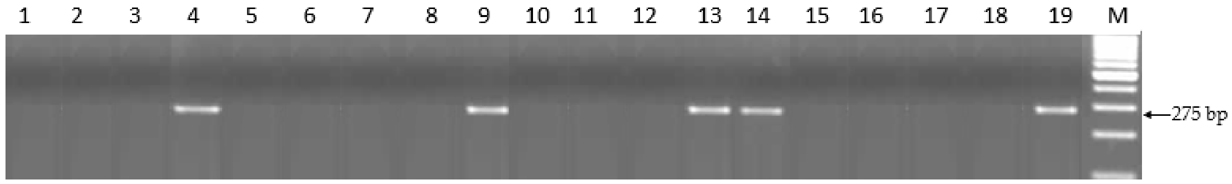

1, Naz/GF55-1; 2, Almaly/GF70; 3, 425/GF55-1; 4, Kupava/ YR5/6/Avocet'S'; 5, Adir/YR2, 6- Sanzar8/BWKLDN9; 7, Viza/Zhenis; 8, 1777Darya/#72Tungysh; 9, 114Novosibirskaya-22/Omskaya37/28; 10, 1777Darya/Tungysh-1; 11, 1777Darya/Tungysh-2; 12, 1777Darya/Tungysh-3; 13, 1777Darya/1724F1-1581/807F4/Naz/Umanka/Almaly/Zimorodok-1; 14, 1777Darya/1724F1-1581/807 F4/Naz /Umanka/Almaly/Zimorodok-2; 15, 12/1613MP-2011/1027/AVS/Ulugbek600/Egemen; 16, 1017/103f3/N91/5353/Egemen-1; 17,1017/103f3/N91/5353/Egemen-2; 18, Avocet'S' (negative control); 19, Avocet S*6/Yr5 (positive control); M – molecular weight marker (Gene-Ruler, 100 bp DNA ladder).

Figure S2. DNA amplification products of wheat entries using primers to the STS S23M41locus linked with the *Yr5* resistance gene. The arrows show the band size of *Yr5*-carrying germplasm (275 bp). The sizes of the bands for *Yr5* are 275 bp (lanes 4, 9, 13, 14 and 19 – positive control). .

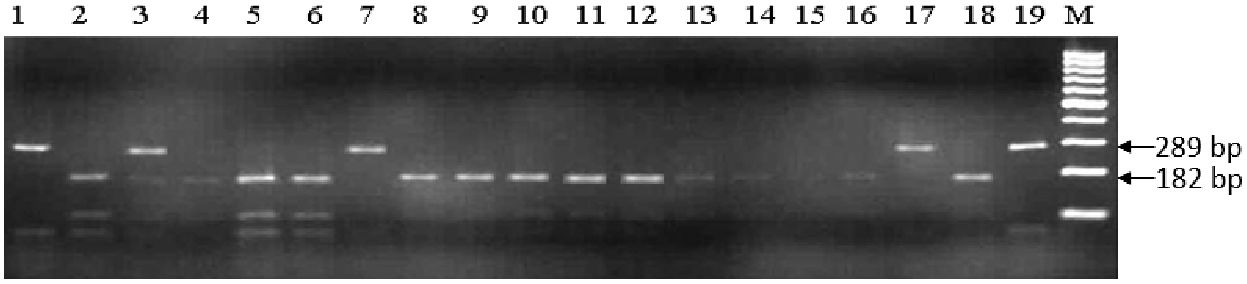

1, Kupava/ YR5/6/Avocet'S'; 2, Adir/YR2; 3, 114Novosibirskaya-22/Omskaya37/28; 4, 1777Darya/Tungysh-1; 5, 1777Darya/Tungysh-2; 6, 1777Darya/Tungysh-3; 7, 1777Darya/1724F1-1581/807F4 /Naz/Umanka/Almaly/ Zimorodok-1; 8, 12/1613MP-2011/1027/AVS/ Ulugbek600 /Egemen; 9, 1017/103f3/N91/5353/Egemen; 10, 1017/103f3/N91/5353/Egemen; 11, 1011/94f3/N23/Knyazhna/Naz-1; 12, 1011/94f3/N23/Knyazhna/Naz-2; 13, 1010/93f3/N23/Kupava/Mereke70-2; 14, Rilsalmaly/Anza; 15, 5-ICARDA-IPBB-2013; 16, 5221/Almaly; 17, 1010/93f3/N23/Kupava/Mereke70-1; 18, Avocet'S' (negative control); 19, Avocet S*6/Yr5 (positive control); M – molecular weight marker (Gene-Ruler, 100 bp DNA ladder).

Figure S3. DNA amplification products of wheat entries using primers to the STS-9/10 locus linked with the *Yr5* re-Scheme 5. carrying (289 bp) and *Yr5*-none-carrying (182 bp) germplasm. PCR products were digested with *Dpn*II; the sizes of the top bands are 289 bp for *Yr5* (lanes 1, 3, 7, 17 and 19– positive control) and 200 bp for non-carriers of *Yr5* (lanes 2-6, 8-16, and 18 – negative control).

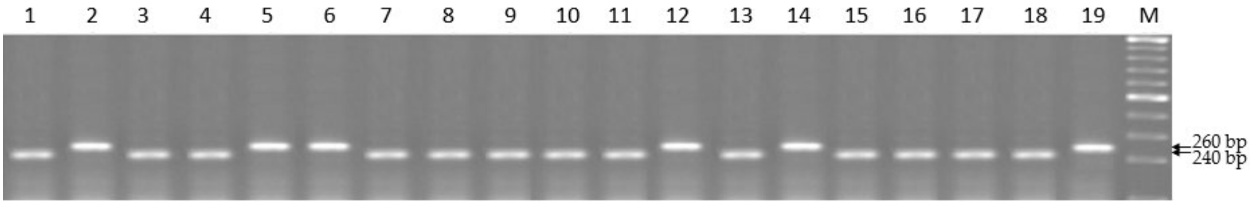

1, Kupava/YR5/6/Avocet'S'; 2, Adir/YR2, 3- Sanzar8/BWKLDN9; 4, 1777Darya/#72Tungysh; 5, Viza/Zhenis; 6, 5-ICARDA-IPBB-2013; 7, 1017/103f3/N91/5353/Egemen-1; 8, 1017/103f3/N91/5353/Egemen-2; 9, 1011/94f3/N23/Knyazhna/Naz-1; 10, 1011/94f3/N23/Knyazhna/Naz-2; 11, 1010/93f3/N23/Kupava/Mereke70-1; 12, Naz/GF55-2, 13- Naz/GF66/Ulugbek600-1; 14, Taza/MK 3750-1; 15, Almaly/GF70/2; 16, #23/Kupava-5; 17, #23/Kupava-10; 18, Avocet'S' (negative control); 19, Avocet S*6/Yr10 (positive control); M -molecular weight marker (Gene-Ruler, 100 bp DNA ladder).

Figure S4. DNA amplification products of wheat entries using primers to the SSR Xpsp3000 locus linked with the *Yr10* resistance gene. The arrows show the band size of *Yr10*-carrying (260 bp) and *Yr10*-none-carrying (240 bp) germplasm. The sizes of the top bands are 260 bp for *Yr10* (lanes 2, 5, 6, 12, 14, and 19– positive control) and 240 bp for non-carriers of *Yr10* (lanes 1, 3, 4, 7-11, 13, 15, 16, and 18 – negative control).

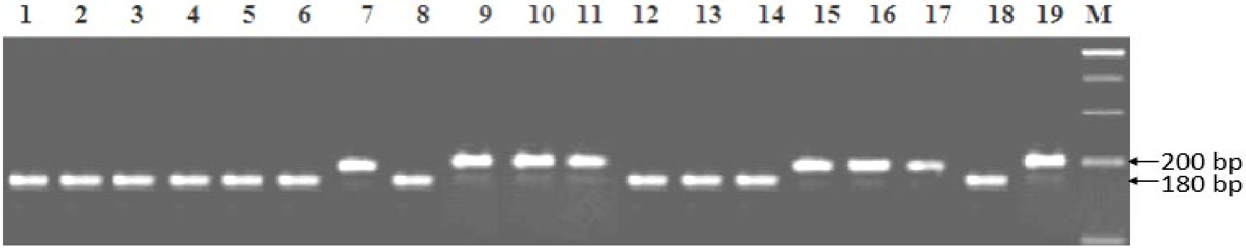

1, Naz/GF55-1; 2, Almaly/GF70; 3, 425/GF55-1; 4, Kupava/YR5/6/Avocet'S'; 5, Sanzar8/BWKLDN9; 6, 1777Darya/#72Tungysh; 7, Viza/Zhenis; 8, 114Novosibirskaya-22/Omskaya37/28; 9, 5-ICARDA-IPBB-2013; 10, Almaly/YR4/Naz; 11, Naz/GF55-2; 12, Naz/GF66/Ulugbek600-1; 13, Naz/GF66/Ulugbek600-2; 14, Naz/Immun78/MK3750; 15, Taza/MK 3750-1; 16, Taza/MK 3750-2; 17, Naz/GF55-3; 18, Avocet'S' (negative control); 19 - Avocet S*6/Yr10 (positive control); M -molecular weight marker (Gene-Ruler, 100 bp DNA ladder).

Figure S5. DNA amplification products of wheat entries using primers to the Yr10SCAR locus linked with the *Yr10* resistance gene. The arrows show the band size of *Yr10*-carrying (200 bp) and *Yr10*-none-carrying (180 bp) germplasm. The sizes of the top bands are 200 bp for *Yr10* (lanes 7, 9, 10, 11, 15, 16, 17, and 19– positive control) and 180 bp for non-carriers of *Yr10* (lanes 1-6, 8, 12, 13, 14, and 18 – negative control).

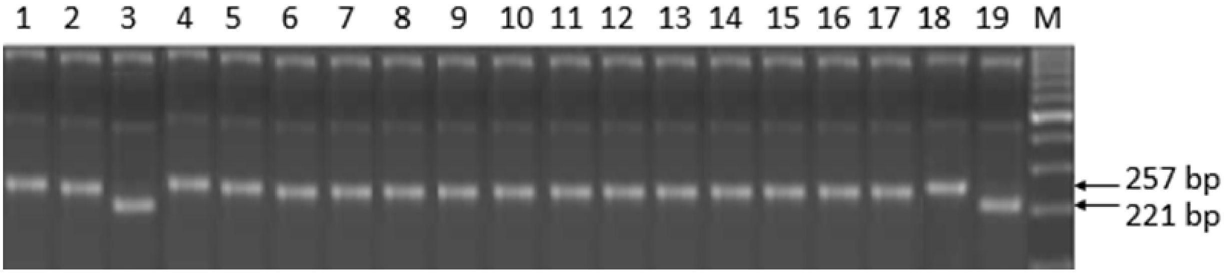

1- Naz/GF55-1, 2- Almaly/GF70, 3- 425/GF55-1, 4- Kupava/ YR5/6/Avocet'S', 5- Adir/YR2, 6- Sanzar8/BWKLDN9, 7- Viza/Zhenis, 8- 1777Darya/#72Tungysh, 9- 114Novosibirskaya-22/Omskaya37/28, 10- 1777Darya/Tungysh-1, 11- 1777Darya/Tungysh-2, 12- 1777Darya/Tungysh-3, 13- 1777Darya/1724F1-1581/807F4 /Naz/Umanka/Almaly/ Zimorodok-1, 14- 1777Darya/1724F1-1581/807 F4/Naz /Umanka/Almaly/Zimorodok-2, 15- 12/1613MP-2011/1027/AVS/ Ulugbek600 /Egemen, 16- 1017/103f3/N91/5353/Egemen-1, 17- 1017/103f3/N91/5353/Egemen-2, 18- Avocet'S' (negative control), 19 - Avocet S*6/Yr15 (positive control), M -molecular weight marker (Gene-Ruler, 100 bp DNA ladder).

Figure S6. DNA amplification products of wheat entries using primers to the SSR Xbarc8 locus linked with the *Yr15* resistance gene. The arrows show the band size of *Yr15*-carrying (221 bp) and *Yr15*-none-carrying (257 bp) germplasm. The sizes of the bands are 221 bp for *Yr10* (lane 3, and 19– positive control) and 257 bp for non-carriers of *Yr15* (lanes 1, 2, 4-17, and 18 – negative control).

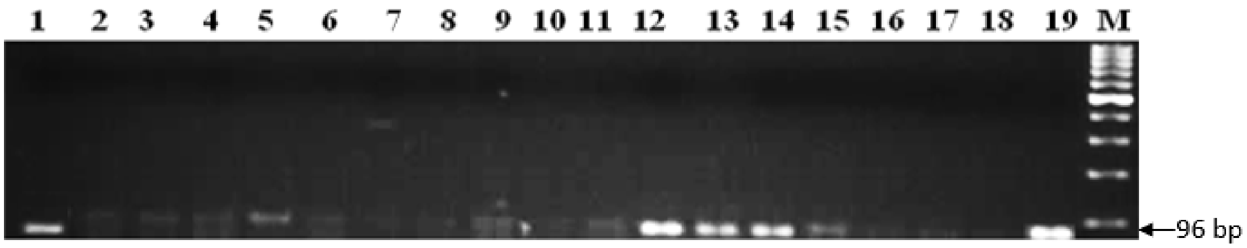

1, RILS-F9 Almaly/Avoset 'S'; 2, Almaly/YR4/Naz; 3, Bogarnaya56/5515/K-47100-Romania; 4, Taza/MK 3750-1; 5, Taza/MK 3750-2; 6, Naz/GF55; 7, Almaly/GF92; 8, 428/MK-122A; 9,Naz/GF55; 10, 425/Renan; 11, 425/GF55; 12, Almaly/GF70/2; 13, #23/Kupava-5; 14, #23/Kupava-7; 15, #23/Kupava-10; 16, #23/Kupava-12; 17, #23/Kupava-16; 18, Avocet'S' (negative control); 19, Avocet S*6/Yr15 (positive control); M -molecular weight marker (Gene-Ruler, 100 bp DNA ladder).

Figure S7. DNA amplification products of wheat entries using primers to the SSR Xgwm413 locus linked with the *Yr15* resistance gene. The arrows show the band size of *Yr15*-carrying (96 bp). The sizes of the bands are 96 bp for *Yr15* (lane 1, 5, 12, 13, 14,15, and 19– positive control).

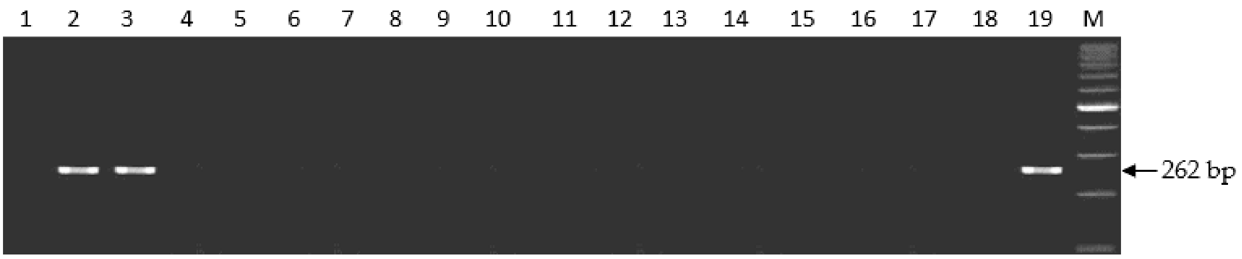

1, Naz/GF55-1; 2,114Novosibirskaya-22/Omskaya37/28; 3, 425/Renan; 4, 1777Darya/Tungysh-1; 5, 1777Darya/Tungysh-2; 6, 1777Darya/Tungysh-3; 7, 1777Darya/1724F1-1581/807F4 /Naz/Umanka/Almaly/ Zimorodok-1; 8, 1777Darya/1724F1-1581/807 F4/Naz /Umanka/Almaly/Zimorodok-2; 9, 12/1613MP-2011/1027/AVS/Ulugbek600/Egemen; 10, 1017/103f3/N91/5353/Egemen-1; 11, 1017/103f3/N91/5353/Egemen-2; 12, 1011/94f3/N23/Knyazhna/Naz-1; 13, 1011/94f3/N23/Knyazhna/Naz-2; 14, 1010/93f3/N23/Kupava/Mereke70-1; 15, 1010/93f3/N23/Kupava/Mereke70-2; 16, Rils Almaly/Anza; 17, 5-ICARDA-IPBB-2013; 18, Avocet'S' (negative control); 19, YR17/LR37/NIL-LR37/TC-6/VPM-RL6081(positive control); M – molecular weight marker (Gene-Ruler, 100 bp DNA ladder).

Figure S8. DNA amplification products of wheat entries using primers to the Ventriup/LN2 locus linked with the *Yr17* resistance gene. The arrows show the band size of *Yr17*-carrying (262 bp). The sizes of the bands are 262 bp for *Yr17* (lane 2, 3, and 19– positive control).

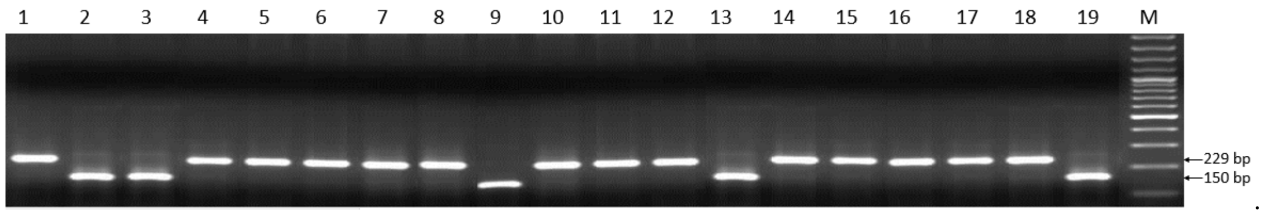

1- Naz/GF55-1, 2- Almaly/GF70, 3- 425/GF55-1, 4- Kupava/ YR5/6/Avocet'S', 5- Adir/YR2, 6- Sanzar8/BWKLDN9, 7- Viza/Zhenis, 8- 1777Darya/#72Tungysh, 9- 114Novosibirskaya-22/Omskaya37/28, 10- 1777Darya/Tungysh-1, 11- 1777Darya/Tungysh-2, 12- 1777Darya/Tungysh-3, 13- 1777Darya/1724F1-1581/807F4 /Naz/Umanka/Almaly/ Zimorodok-1, 14- 1777Darya/1724F1-1581/807 F4/Naz /Umanka/Almaly/Zimorodok-2, 15- 12/1613MP-2011/1027/AVS/ Ulugbek600 /Egemen, 16- 1017/103f3/N91/5353/Egemen-1, 17- 1017/103f3/N91/5353/Egemen-2, 18- Avocet'S' (negative control), 19 - YR18/NIL-LR34/TC-6/PI58548 (positive control), M -molecular weight marker (Gene-Ruler, 100 bp DNA ladder).

Figure S9. DNA amplification products of wheat entries using primers to the STS csLV34 locus linked with the *Yr18*/*Lr34* resistance gene. The arrows show the band size of *Yr18* -carrying (150 bp). The sizes of the bands are 229 bp for *Yr17* (lane 2, 3, and 19– positive control).
